# Malformations of Human Neocortex in Development – Their Progenitor Cell Basis and Experimental Model Systems

**DOI:** 10.3389/fncel.2019.00305

**Published:** 2019-07-09

**Authors:** Anneline Pinson, Takashi Namba, Wieland B. Huttner

**Affiliations:** Max Planck Institute of Molecular Cell Biology and Genetics, Dresden, Germany

**Keywords:** neocortex, malformations, development, progenitors, model systems

## Abstract

Malformations of the human neocortex in development constitute a heterogeneous group of complex disorders, resulting in pathologies such as intellectual disability and abnormal neurological/psychiatric conditions such as epilepsy or autism. Advances in genomic sequencing and genetic techniques have allowed major breakthroughs in the field, revealing the molecular basis of several of these malformations. Here, we focus on those malformations of the human neocortex, notably microcephaly, and macrocephaly, where an underlying basis has been established at the level of the neural stem/progenitor cells (NPCs) from which neurons are directly or indirectly derived. Particular emphasis is placed on NPC cell biology and NPC markers. A second focus of this review is on experimental model systems used to dissect the underlying mechanisms of malformations of the human neocortex in development at the cellular and molecular level. The most commonly used model system have been genetically modified mice. However, although basic features of neocortical development are conserved across the various mammalian species, some important differences between mouse and human exist. These pertain to the abundance of specific NPC types and/or their proliferative capacity, as exemplified in the case of basal radial glia. These differences limit the ability of mouse models to fully recapitulate the phenotypes of malformations of the human neocortex. For this reason, additional experimental model systems, notably the ferret, non-human primates and cerebral organoids, have recently emerged as alternatives and shown to be of increasing relevance. It is therefore important to consider the benefits and limitations of each of these model systems for studying malformations of the human neocortex in development.

## Introduction

Malformations of the human cerebral cortex (e.g., microcephaly, megalencephaly, lissencephaly, focal cortical dysplasia, polymicrogyria), notably of the neocortex, represent an important cause of intellectual disability and of neurological as well as psychiatric disorders such as epilepsy and autism (Juric-Sekhar and Hevner, 2019). Of these cortical malformations, microcephaly and megalencephaly are thought to be primarily caused by alterations in NPC proliferation, abundance and function, whereas the other types of malformations have been shown to be largely caused by alterations in neuronal migration (Barkovich et al., 2012; Juric-Sekhar and Hevner, 2019).

Mutations in a number of identified genes have been found to underlie autosomal recessive primary microcephaly (MCPH) (e.g., *MCPH1, ASPM, CASC5*, and *WDR62*) and thanatophoric dysplasia (*FGFR3*), a subtype of megalencephaly (Jayaraman et al., 2018; Juric-Sekhar and Hevner, 2019). However, not only gene mutations can cause neocortical malformations, but also external factors such as viral infections. The latter is notably the case for Zika virus, which induces microcephaly (Mlakar et al., 2016; Oliveira Melo et al., 2016). Researchers have made major efforts to understand the causality of the mutations and the molecular functions of these genes, as well as the effects of the viral infections, using various experimental model organisms (Inglis-Broadgate et al., 2005; Lizarraga et al., 2010; Pulvers et al., 2010; Gruber et al., 2011; Cugola et al., 2016; Jayaraman et al., 2016; Ding et al., 2019). Because basic features of neocortical development are conserved across mammals, notably from mouse to human, genetically modified mice have served as the most commonly used model organism. However, several important differences in neocortical development between mouse and human have been identified over the last decade, as is discussed below. Therefore, researchers have started to explore alternative model systems that more faithfully recapitulate the phenotypes of human neocortical malformations. These include gyrencephalic model animals that are relatively amenable, notably the ferret, non-human primates (especially marmoset and macaque), and human iPSC-derived cerebral organoids.

The advancements in neuroimaging techniques, genome editing, and single-cell manipulations over the last decade have allowed clinical and basic researchers to not only discover novel types of neocortical malformations but also elucidate their underlying mechanisms. In the context of microcephalic and megalocephalic malformations, NPCs are crucial cell populations to study as any disruption of their tightly controlled behavior could affect many aspects of neocortical development. In particular, a better understanding of the cell biology of NPCs under physiological conditions is essential to dissect their roles in pathological conditions. In this article, we focus on NPCs and review their main characteristics that could potentially be affected in neocortical malformations. We also provide information how to study NPCs in this context. In addition, we discuss the advantages and limitations of different experimental model systems that are being used to recapitulate, as faithfully as possible, the phenotype of the human malformation under study.

## NPC Types, Cell Biology, and Markers: Their Relevance for Studying Malformations of the Human Neocortex in Development

### Cytoarchitecture of the Developing Neocortex

Most basic principles of the cytoarchitecture of the developing neocortex are similar for mouse, ferret, non-human primates, and human (Götz and Huttner, 2005; Lui et al., 2011; Taverna et al., 2014; Dehay et al., 2015; Fernandez et al., 2016; Namba and Huttner, 2017). The developing neocortex has overt tissue polarity. Specifically, the apical side is the ventricular surface and the basal side is the pial surface. Typically, there are four major zones at mid-neurogenesis, which are – from apical to basal – the ventricular zone (VZ), the subventricular zone (SVZ), the intermediate zone (IZ), and the cortical plate (CP) (flanked by subplate and marginal zone).

**FIGURE 1:**
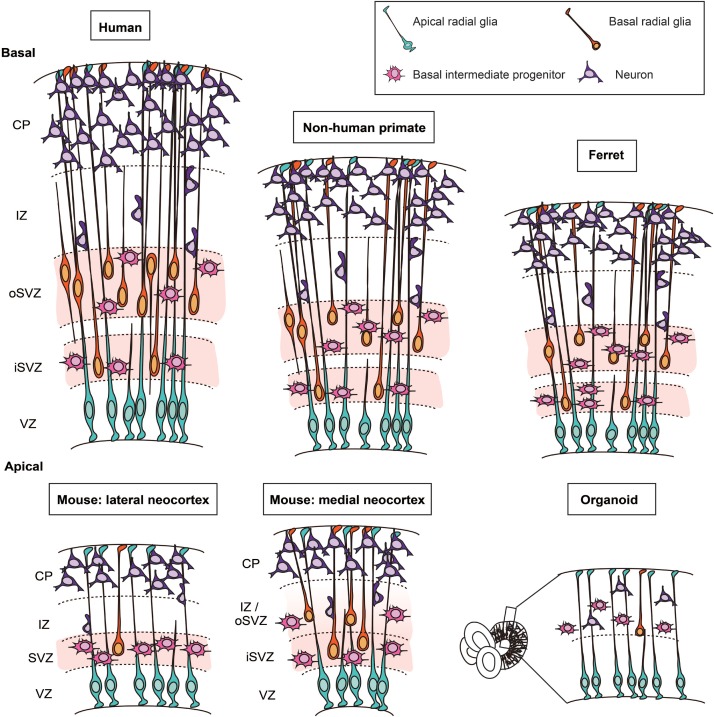
Cytoarchitecture and cell types in the developing neocortex of human, non-human primate, ferret, mouse, and in cerebral organoids. Schematic representation of the cytoarchitecture of the developing neocortex. The developing neocortex is divided into different layers. On the apical side, the germinal zone lining the ventricle, the ventricular zone (VZ), harbors the cell bodies of apical radial glia. In ferret, non-human primates and human, the subventricular zone (SVZ) is divided into two sublayers: the inner SVZ (iSVZ) and the outer SVZ (oSVZ). The SVZ is populated by two main types of basal progenitors (i) basal intermediate progenitors and (ii) basal radial glia. Basal radial glia only represent a small fraction of the basal progenitors in the embryonic mouse neocortex. In human cerebral organoids, which are derived from human induced pluripotent stem cells or human embryonic stem cells, apical radial glia are located in the VZ. The basal progenitors and neurons are located on the basal side.

A prominent difference between mouse, ferret, non-human primates, and human has been observed with regard to the SVZ (Smart et al., 2002; Fietz et al., 2010; Hansen et al., 2010). In the early phase of neurogenesis, the relative thickness of the SVZ is not strikingly different across these species. As neurogenesis proceeds, the SVZ of developing ferret, non-human primate and human neocortex massively grows in radial thickness and splits into an inner SVZ (iSVZ) and an outer SVZ (oSVZ). This split was first described for the developing macaque neocortex (Smart et al., 2002; Figure 1). The growth in SVZ thickness pertains predominantly to the oSVZ and reflects an increase in the pool size of the so-called BPs, the class of NPCs implicated in the expansion of the neocortex, as is delineated below.

### NPC Types and Their Cell Biology

Before the onset of neurogenesis, the neural tube – also in its anterior domain, which gives rise to the neocortex – is composed of a monolayer of neural stem cells, the neuroepithelial cells (NECs). Although a monolayer of cells, the neuroepithelium appears stratified, a feature referred to as pseudostratification (Götz and Huttner, 2005). This pseudostratification is due to the movements of the NEC nucleus along the apical-basal axis of the neuroepithelium during the cell cycle, a process called interkinetic nuclear migration (INM) (Sauer, 1935; Taverna et al., 2014). Specifically, NECs characteristically divide at the ventricular surface, move their nucleus basally during G1 for S-phase in the basal region of the neuroepithelium (i.e., near the basal lamina), followed by apically directed migration of the nucleus back to the ventricular surface during G2 for the next mitosis (Taverna et al., 2014).

Neuroepithelial cells initially self-expand by symmetric proliferative divisions. With the onset of neurogenesis, NECs switch to differentiative asymmetric cell divisions. At this stage of cortical development, NECs transform into apical (or ventricular) radial glia (aRG) (Götz and Huttner, 2005). An asymmetric NEC division gives rise to an aRG daughter cell and, typical for the mammalian neocortex, a BP daughter cell, a process referred to as indirect neurogenesis. Rarely in the developing neocortex of mammals, in contrast to the developing spinal cord or to the developing brains of non-mammalian vertebrates, the second daughter cell is a neuron, a process referred to as direct neurogenesis (Cardenas et al., 2018). Of relevance for studying malformations of the human neocortex in development, and consistent with observations reported, both (i) a premature switch of NECs to differentiative asymmetric cell divisions and (ii) an increase in direct neurogenesis at the expense of indirect neurogenesis, should be considered as potential causes of microcephaly, as both would eventually result in reduced neuron numbers.

#### aRG

The cell bodies of aRG are located in the VZ. As the neuroepithelium transforms from a monolayer into a tissue with various zones (see above) with the onset of neurogenesis, aRG maintain contact with the basal lamina by extending a basal process, also referred to as radial fiber, through the SVZ, IZ, and CP. Hence, like NECs, aRG maintain epithelial features (e.g., apical-basal polarity with basal lamina contact and apical junctions) and also show INM, which however is confined to the VZ (Götz and Huttner, 2005; Lui et al., 2011; Taverna et al., 2014; Dehay et al., 2015; Fernandez et al., 2016; Namba and Huttner, 2017). aRG gradually switch their mode of division from self-amplifying (symmetric proliferative) to asymmetric differentiative division that results in their self-renewal and typically the generation of a BP (rarely of a neuron) (Malatesta et al., 2000; Miyata et al., 2001; Noctor et al., 2001; Tamamaki et al., 2001; Götz and Huttner, 2005). Similar to the considerations pertaining to NECs mentioned above, both (i) a premature switch of aRG to differentiative asymmetric cell divisions and (ii) an increase in direct neurogenesis by aRG at the expense of indirect neurogenesis, should be considered as potential causes of microcephaly.

In fetal human neocortex, aRG shorten their basal process and thus lose basal lamina contact around gestational week (GW) 17, becoming so-called truncated RG (Nowakowski et al., 2016b). aRG and truncated RG differ not only in their morphology but also in their gene expression patterns (see below). aRG also play another important role, by providing their basal process as a scaffold for neuronal migration (Rakic, 1972). In the present review, we will not address malformations of the human neocortex that are caused by defective neuronal migration, but rather refer the reader to excellent reviews on this topic (Rakic, 2003a,b; Reiner, 2013; Romero et al., 2018; Buchsbaum and Cappello, 2019).

#### BP Types

Two main types of BPs have been described, basal intermediate progenitors (bIPs) and basal (or outer) radial glia (bRG). bIPs lack apical-basal cell polarity, exhibiting multiple short processes in interphase that are retracted for mitosis (Götz and Huttner, 2005; Attardo et al., 2008; Lui et al., 2011; Taverna et al., 2014; Dehay et al., 2015; Fernandez et al., 2016; Namba and Huttner, 2017). bIPs can be either proliferative or neurogenic, with striking differences in the abundance of these two bIP subtypes across mammals (Florio and Huttner, 2014). Proliferative bIPs undergo symmetric proliferative divisions, which increase their pool size, before turning into neurogenic bIPs that give rise to two post-mitotic neurons in a final, consumptive division (Haubensak et al., 2004; Miyata et al., 2004; Noctor et al., 2004; Hansen et al., 2010; Lui et al., 2011). Proliferative bIPs are a major fraction of bIPs in fetal human neocortex, where bIPs constitute about half of all BPs, whereas neurogenic bIPs represent the main BPs in the developing mouse neocortex (Haubensak et al., 2004; Miyata et al., 2004; Noctor et al., 2004; Wu et al., 2005; Attardo et al., 2008; Betizeau et al., 2013). Prior to the characterization of bRG, proliferative bIPs were considered as key for neocortical expansion in an insightful Perspectives article (Kriegstein et al., 2006). We would like to emphasize that even though bRG with their proliferative capacity have emerged, over the past decade, as a major NPC type underlying neocortical expansion, one should not underestimate the role of proliferative bIPs in this process. Hence, it should be born in mind that impairment of the proliferative capacity of bIPs may constitute a cause underlying malformations of the human neocortex in development, notably microcephaly.

Basal radial glia were originally characterized in developing gyrencephalic neocortex, notably of human and ferret (Fietz et al., 2010; Hansen et al., 2010; Reillo et al., 2011). bRG have subsequently also been found at low abundance in embryonic mouse lateral neocortex (Shitamukai et al., 2011; Wang et al., 2011), and in embryonic mouse medial neocortex at higher abundance, similar to human and ferret developing neocortex (Vaid et al., 2018) (see below for using the embryonic mouse medial neocortex as model system). There are several morphotypes of bRG. The “classical” morphotype, which is conserved from rodents to human, extends a basal process toward the basal lamina. In addition, recent studies revealed that the morphological heterogeneity of bRG is greater in developing gyrencephalic than rodent neocortex (Betizeau et al., 2013; Reillo et al., 2017; Kalebic et al., 2019). These additional bRG morphotypes exhibit an apical process and/or one or two basal processes (Betizeau et al., 2013; Reillo et al., 2017; Kalebic et al., 2019). The increase in the number of processes in human bRG has been shown to be causally linked to their greater proliferative capacity and has been attributed to expression of the morpho-regulatory protein PALMDELPHIN (Kalebic et al., 2019). Given that the proliferative capacity of bRG is thought to be a crucial parameter for neocortical expansion, these data raise the possibility that alterations in the extent of bRG process growth may contribute to malformations of the human neocortex in development.

Basal radial glia show a characteristic, typically basally directed, movement of their cell body, including nuclear material, during M-phase, which has been referred to as mitotic somal translocation (Hansen et al., 2010; Lui et al., 2011; Betizeau et al., 2013; LaMonica et al., 2013; Ostrem et al., 2014). This movement is driven by actomyosin contractility, as is the case for INM of aRG (Schenk et al., 2009). Because the bRG cell bodies move more basally within the oSVZ at every cell division by mitotic somal translocation, this movement might be involved in the radial expansion of the oSVZ during corticogenesis, which in turn is thought to be a hallmark of neocortical expansion.

In primates including human, self-renewing divisions of bRG occur throughout the neurogenic period, maintaining the bRG pool size at an appropriate level for neuron production (Betizeau et al., 2013). This is likely to be crucial in particular for the late stages of neocortical neurogenesis, when upper-layer neurons are produced, as an increase in upper-layer neuron production is a characteristic feature of the evolutionary expansion of the neocortex (Kriegstein et al., 2006). Hence, malformations of the human neocortex associated with a relative decrease in upper-layer neuron abundance may be caused by a decrease in bRG proliferative or self-renewal capacity in the course of neocortical neurogenesis.

#### The Cell Biological Advantage of BPs for Maximizing NPC Division

Like in the case of NECs, aRG mitoses are confined to the ventricular surface. This reflects their epithelial nature. Specifically, aRG extend a primary cilium into the ventricular fluid, an organelle that persists throughout interphase and tethers the centrosomes to the apical cell cortex (Paridaen and Huttner, 2014; Wilsch-Bräuninger et al., 2016). Hence, one role of the INM of aRG is to enable the nucleus to be in the vicinity of the centrosomes at the onset of mitosis, as the primary cilium is dismantled/endocytosed, allowing the two centrosomes to become mitotic spindle poles (Taverna et al., 2014). However, the size of the ventricular surface limits the number of aRG mitoses. Hence, a second role of INM is to move the interphase nuclei of aRG away from the ventricular surface in order to increase the space for apical mitoses (Smart, 1972a,b; Taverna et al., 2014). Yet, even with INM, the limited size of the ventricular surface still puts a constraint on the number of aRG mitoses.

In contrast to aRG, BPs no longer exhibit apical cell polarity, that is, their primary cilium does not extend from the apical but from the basolateral plasma membrane, and they are no longer integrated into the apical junctional belt but have delaminated from the ventricular surface at mitosis (Wilsch-Bräuninger et al., 2012). These differences in the cell biology of BPs in comparison to aRG constitute a fundamental advantage with regard to maximizing their mitoses. Along with the delamination process, the BP centrosomes adopt a perinuclear localization (Taverna et al., 2016; Wilsch-Bräuninger et al., 2016), and so BPs can undergo mitosis essentially anywhere in the SVZ, thereby massively increasing their pool size and, if required, the radial thickness of the SVZ, notably of the oSVZ. In other words, the loss of apical cell polarity and, consequently, of a major spatial limitation for cell division, is at the core of BPs evolving to become the pivotal NPC class for neocortical expansion. It follows from these considerations that any impairment of BP abundance and/or function is likely to result in microcephaly.

### NPC Markers

To investigate the role of NPCs in malformations of the human neocortex in development, one needs to identify the various NPC types described above in the experimental model system under study. To this end, several criteria can be used, such as the localization of the cell body of NPCs at mitosis, NPC morphology at mitosis or in interphase, and notably marker expression. The location of the cell body at mitosis is a useful criterion to distinguish aRG and BPs (Fietz and Huttner, 2011). Using markers of mitotic cells, typically phosphorylated histone H3 or phosphorylated vimentin (Kamei et al., 1998), one can distinguish aRG that undergo mitosis at the apical surface, and BPs that undergo mitosis away from the apical surface (by definition more than three nuclear diameter, > 30 μm), typically in the SVZ.

The morphology of BPs at mitosis is useful to distinguish bIPs and bRG. Because vimentin is a cytoplasmic intermediate filament protein, immunostaining for phosphorylated vimentin can be used to visualize cell morphology at mitosis with regard to the absence (bIPs) or presence (bRG) of cell processes, which is not possible using phosphorylated histone H3 immunostaining. Another intermediate filament protein that is a radial glia (RG) marker, nestin (Frederiksen and McKay, 1988), can also be used for distinguishing mitotic bRG with their process(es) from mitotic bIPs (Fietz et al., 2010).

Regarding aRG and bRG in interphase, addition of a lipophilic dye such as DiI to either the basal or apical side of the cortical wall can be used to identify aRG and bRG by virtue of their plasma membranes contacting the basal lamina (aRG, bRG) and the ventricular surface (aRG). As the added dye diffuses in the plane of the plasma membrane, it outlines the cell shape in interphase with the basal (aRG, bRG) and apical (aRG) cell process (Miyata et al., 2001; Fietz et al., 2010; Hansen et al., 2010; Taverna et al., 2016). To visualize the cell morphology in interphase not only of aRG and bRG but also of bIPs, membrane-targeted fluorescent proteins (e.g., LynGFP, GFP-CAAX) expressed by electroporation (Okamoto et al., 2013; Kalebic et al., 2019) or viral vectors (Noctor et al., 2001) have been used.

Neural stem/progenitor cell markers are instrumental to identify NPC types and to study their potential role in malformations of the human neocortex in development. NPC marker expression patterns have mostly been studied in embryonic mouse neocortex. However, one has to be aware that the expression patterns may be different in other mammalian species, and even in the same species may vary between developmental stages and neocortical regions. Moreover, the expression pattern of a given NPC marker is not necessarily identical when examining its mRNA by *in situ* hybridization of tissue or RNA sequencing/quantification of isolated cells vs. determining its protein immunoreactivity by immunohistochemistry. A striking case of such disparity is Tbr2, the mRNA of which but not the protein is found in aRG (Florio et al., 2015).

#### aRG and bRG Markers

Most of the RG markers are expressed in both aRG and bRG in any part of the neocortex throughout the developmental period. Proteins used to detect RG in various mammalian species include nestin (Frederiksen and McKay, 1988), vimentin (Bignami et al., 1982), GLAST (Shibata et al., 1997), BLBP (Feng et al., 1994; Kurtz et al., 1994), Sox2 (Bani-Yaghoub et al., 2006), and Pax6 (Götz et al., 1998). Note that some of these classical RG “marker” proteins (e.g., Sox2) have also been found to be expressed in bIPs in both embryonic mouse and fetal human neocortex, albeit typically at lower levels than in RG (Table 1).

**Table 1:** Progenitor cell classes, types, and markers.

	Human aRG < GW17	Human aRG > GW17	Mouse aRG	Human bRG	Mouse bRG lateral	Mouse bRG medial	Human bIPs	Mouse bIPs
Nestin	+ (28)	+ (7)	+ (11, 18)	+ (7)	+ (25)	ND (mRNA+) (23)	+/− (7)	+/− (5)
GLAST	+ (28)	+ (7)	+ (11)	+ (7)	+ (25)	ND (mRNA+) (23)	+/− (7, 15)	+/− (15)
Vimentin	+ (7, 28)	+ (13)	+ (16)	+ (7)	+ (24)	+ (23)	+/− (16)	+/− (5)
BLBP	+ (7, 19)	+ (2)	+ (6)	+ (16)	+ (12)	ND (mRNA+) (23)	+/− (16)	+/− (27)
Sox2	+ (10)	+ (10)	+ (1)	+ (10)	+ (24)	+ (23)	+/− (16)	+/− (14)
Pax6	+ (7,10)	+ (7)	+ (9)	+ (7, 10)	+ (24)	+ (23)	+/− (7)	+/− (5)
CRYAB	ND (mRNA+) (8)	+ (20, 22)	ND (mRNA+) (8)	− (20, 22)	ND (mRNA+) (8)	ND (mRNA+) (23)	ND (mRNA−) (21)	ND (mRNA+) (8)
ANXA1	ND (mRNA+) (8)	+ (22)	ND (mRNA+) (8)	− (22)	ND (mRNA+) (8)	ND (mRNA−) (23)	+/− (22) (18)	ND (mRNA+) (8)
GFAP	+ (3)	+ (7)	− (27)	+ (7)	ND (mRNA−) (8)	ND (mRNA+) (23)	ND (mRNA−) (21)	− (17)
Hopx	+ (20)	− (20)	+ (23)	+ (20, 21, 22)	− (20)	+ (23)	+/− (16,21)	− (23)
PTPRZ1	+ (21)	− (21)	+ (21)	+ (21)	+ (21)	ND (mRNA+) (23)	− (21)	− (21)
TNC	+ (21)	− (21)	+ (21)	+ (21)	+ (21)	ND (mRNA+) (23)	− (21)	− (21)
Tbr2	− (7,10)	− (4,7)	− (5)	− (7,10) (mRNA+)	+/− (8, 24)	+/− (23)	+ (8, 21)	+ (5)

*Expression of markers in human and mouse apical radial glia cells, basal radial glia cells, and basal intermediate progenitors cells. +, the protein is expressed; −, the protein is not expressed; ND, no data is available for the protein expression. When there is no data available for the protein expression, the mRNA expression is added (mRNA+, mRNA−). (1) Bani-Yaghoub et al. (2006). (2) Brochner and Mollgard (2016). (3) Choi and Lapham (1978). (4) Choi (1986). (5) Englund et al. (2005). (6) Feng et al. (1994). (7) Fietz et al. (2010). (8) Florio et al. (2015). (9) Götz et al. (1998). (10) Hansen et al. (2010). (11) Hartfuss et al. (2001). (12) Heng et al. (2017). (13) Howard et al. (2008). (14) Hutton and Pevny (2011). (15) Johnson et al. (2015). (16) Kalebic et al. (2019). (17) Kowalczyk et al. (2009). (18) Mission et al. (1991). (19) Mo and Zecevic (2007). (20) Nowakowski et al. (2016b). (21) Pollen et al. (2015). (22) Thomsen et al. (2016). (23) Vaid et al. (2018). (24) Wang et al. (2011). (25) Wang et al. (2016). (26) Woodhams et al. (1981). (27) Wu et al. (2016). (28) Zecevic (2004).*

Recent advances in single-cell RNA sequencing have led to the identification of several new RG markers (Pollen et al., 2015; Thomsen et al., 2016). For example, Hopx, PTPRZ1, and TNC (tenascin-C) are selectively expressed in bRG at later stages of human neocortical neurogenesis (after GW17), but before GW17, these molecules are also expressed in aRG. In contrast, the newly identified aRG markers CRYAB and ANXA1 are specifically found in aRG, but neither in bRG nor bIPs (Table 1; Thomsen et al., 2016).

Hopx has become a widely used marker for RG in the developing human (Pollen et al., 2015; Nowakowski et al., 2016b; Thomsen et al., 2016) and ferret (Vaid et al., 2018) neocortex. In embryonic mouse lateral neocortex, Hopx is expressed in aRG at early stages, thereafter the expression gradually decreases. In contrast, Hopx expression becomes prominent in the mouse medial neocortex around E17, and there, Hopx is also expressed in bRG (Vaid et al., 2018) (see below for using the embryonic mouse medial neocortex as model system). Human and non-human primate RG express GFAP, but mouse RG in the embryonic lateral neocortex do not (reviewed in Howard et al., 2008; Table 1).

#### bIP Markers

The most commonly used marker for bIPs is Tbr2 (*EOMES*) (Englund et al., 2005; Hevner, 2019). In fetal human neocortex, the Tbr2 protein is mostly expressed in bIPs, but its mRNA (*EOMES*) is also found in RG (Florio et al., 2015; Johnson et al., 2015). The disparity between Tbr2 mRNA and protein expression reflects a microRNA-mediated inhibition of Tbr2 mRNA translation in aRG (Florio et al., 2015). In embryonic mouse neocortex, Tbr2 protein expression is detectable in both bIPs and bRG (in contrast to human (Fietz et al., 2010; Florio et al., 2015; Table 1). Similarly, Tbr2 protein is also expressed in a subset of non-human primate bRG (Betizeau et al., 2013).

## Experimental Models for Studying Human Neocortical Malformations: Their Advantages and Limitations

### Mouse

Mouse is the most frequently used model organism to study human neocortical malformations. The big advantages of mouse as a model organism are (i) a relatively short life cycle and (ii) relatively easy and established ways to manipulate the expression of a given gene under study. Primary microcephaly is a developmental disorder characterized by a smaller head and brain, which is typically due to a reduced proliferation of NPCs (Jayaraman et al., 2018). Several gene mutations in human have been associated with MCPH, including *MCPH1, WDR62, CDK5RAP2, ASPM, CENPJ*, and *CEP63* (Jayaraman et al., 2018).

For several gene mutations, KO mice nicely recapitulate the human phenotype. One example is *MCPH1*, encoding the protein microcephalin, which was the first gene identified to be associated with primary microcephaly (Jackson et al., 2002). *MCPH1* mutant mice faithfully recapitulate key features of the primary microcephaly observed in human patients. In the KO mouse model, the size of the brain was overtly smaller (Gruber et al., 2011; Zhou et al., 2013). Moreover, the KO mouse has allowed the identification of the underlying mechanism, a premature switch from symmetric proliferative to asymmetric differentiative aRG division, preventing the normally occurring increase in the aRG pool size and, consequently, reducing neuron production (Gruber et al., 2011). This switch to premature asymmetric differentiative aRG division is brought about by a change in the alignment of the mitotic spindle, and hence in cleavage plane orientation (Gruber et al., 2011), an established cause for such a switch.

The second example is *WDR62* (WD repeat-containing protein 62). Mutations in human WDR62 induce a broad spectrum of cortical malformations, including primary microcephaly, pachygyria (reduction in the number of gyri, which however are unusually thick), and hypoplasia of the corpus callosum (Bilguvar et al., 2010; Yu et al., 2010). The Wdr62 KO mouse recapitulates the primary microcephaly observed in human (Jayaraman et al., 2016), but not the pachygyria. The microcephaly appears to be due to a defect in centriole duplication and a premature delamination of aRG from the VZ which then become precociously differentiating BPs (Jayaraman et al., 2016). Like WDR62, CENPJ (centrosome protein J), also known as SAS-4, is required for centriole biogenesis in both mouse and human, providing a likely explanation for the primary microcephaly observed upon mutation in the human *CENPJ* gene (Insolera et al., 2014; Ding et al., 2019).

The third example is *CDK5RAP2*, which encodes the CDK5 Regulatory Subunit Associated Protein 2 (CDK5RAP2). In *CDK5RAP2* mutant mice, the brain is overtly smaller, and upper-layer neurons are reduced. This appears to be predominantly due to premature cell cycle exit of BPs and increased apoptosis (Lizarraga et al., 2010).

The last example we wish to discuss is *CEP63*, which encodes centrosomal protein 63. Mutations in human *CEP63* induce Seckel syndrome, which is characterized by primary microcephaly. *CEP63* KO mice recapitulate the human microcephalic phenotype. The microcephaly is due to p53-dependent cell death of NPCs triggered by centrosome-based mitotic errors (Marjanovic et al., 2015).

Neural stem/progenitor cells also play important roles in macrocephalic pathophysiology (Juric-Sekhar and Hevner, 2019). Hemimegalencephaly, a neurological malformation in which one side of the brain is abnormally large, has been linked to an overactivation of the mTOR signaling pathway in NPCs (Lee et al., 2012; Poduri et al., 2012). Specifically, *de novo* somatic mutations in human genes of the mTOR pathway such as *PI3K* or *CCND2*, which result in such overactivation, have been shown to cause hemimegalencephaly, and this megalencephaly has been successfully modeled in mouse (Mirzaa et al., 2014; Roy et al., 2015; D’Gama et al., 2017).

In addition to these genetic mutations, mouse can be used to recapitulate neocortical malformations induced by external factors such as alcohol or viral infections. This is notably the case for the microcephaly caused by Zika virus infection, which has been reproduced in mouse models (Cugola et al., 2016; Nowakowski et al., 2016a; Gladwyn-Ng et al., 2018).

Whereas the above examples document the advantages of the mouse model system for studying human neocortical malformations, this system does have its limitations, as evidenced for another gene involved in human MCPH. Thus, mutations in *ASPM* (*abnormal spindle-like microcephaly-associated*), the gene which is most frequently affected in primary microcephaly, drastically reduce cortical volume in human (Desir et al., 2008; Passemard et al., 2016). In contrast, *Aspm* mutant mice exhibit only mild microcephaly (Pulvers et al., 2010; Fujimori et al., 2014; Jayaraman et al., 2016), which therefore represents a case in which the mouse model shows its limitations for studying cortical malformations. However, a more severe phenotype has been reported for another *Aspm* KO mouse (Capecchi and Pozner, 2015). This raises the question what the variability in the severity of the microcephalic phenotype between the various *Aspm* mutant mice, and the phenotypic differences between *Aspm* mutant mice and humans with *ASPM* mutations, might reflect. In considering possible answers, in particular regarding the latter issue, it should be realized that the ratio of *Aspm* expression in mouse SVZ/bIPS/bRG over VZ/aRG is lower than that for *ASPM* in human iSVZ/oSVZ/bRG over VZ/aRG (Fietz et al., 2012; Florio et al., 2015).

There are additional limitations of the mouse model that should be taken into consideration. First, as mentioned above, the cytoarchitecture of the mouse and human developing neocortex exhibits a major difference as the human SVZ is profoundly enlarged and divided into two subzones, the iSVZ and the oSVZ (Smart et al., 2002; Figure 1). Second, human BPs are highly proliferative cells whereas mouse BPs, which are mostly bIPs, typically divide only once giving rise to two neurons (Haubensak et al., 2004; Miyata et al., 2004; Noctor et al., 2004). Third, another major difference is the abundance of bRG, which is high in fetal human neocortex but low in embryonic mouse lateral neocortex. In this context, a recent study has shown that the abundance of bRG in mouse medial neocortex at later stages of embryonic development is similar to that in developing gyrencephalic neocortex (Vaid et al., 2018). Moreover, the gene expression profile of the mouse medial bRG was found to be closer to that of human bRG than that of mouse lateral bRG (Vaid et al., 2018). This raises the possibility that the embryonic mouse medial neocortex may be a more appropriate target tissue than the embryonic mouse lateral neocortex when phenotypes of human cortical malformations that are likely to pertain to the proliferative capacity of bRG are to be analyzed. Fourth, although not a focus of this review, it is important to realize that lissencephaly, a cortical malformation largely due to defects in neuronal migration, would be impossible to model in mouse because this mammal has a lissencephalic neocortex by nature. These considerations highlight the limitations of the mouse model and the importance of choosing the right model system when studying a human neocortical malformation.

### Ferret

Ferrets are carnivores and belong to the category of gyrencephalic mammals. While presenting some limitations (specified below), they exhibit more similarities with developing human neocortex than mouse does. First, the cytoarchitecture of the ferret neocortex is characterized by an SVZ that, as in human, is divided into an iSVZ and an oSVZ (Fietz et al., 2010; Reillo et al., 2011; Figure 1). Second, bRG are present in the developing ferret neocortex at much greater abundance than in the mouse lateral neocortex (Reillo et al., 2011), and their morphology resembles that of human bRG (Kalebic et al., 2019).

Of note, whereas the phenotypes of certain cortical malformations are not fully recapitulated in embryonic mouse neocortex, they can be in developing ferret neocortex. Specifically, the adaptation of the *in utero* electroporation technique, which has been commonly used in embryonic mouse neocortex, to the developing ferret neocortex (Kawasaki et al., 2012, 2013) has allowed researchers to recapitulate the phenotype of a cortical malformation known as thanatophoric dysplasia (TD) (Masuda et al., 2015). TD is a skeletal dysplasia characterized by several abnormalities, including polymicrogyria, megalencephaly, and subarachnoid and subependymal heterotopia. It is caused by a mutation in the *fibroblast growth factor receptor 3* gene leading to continuous activation of the receptor (Rousseau et al., 1994; Shiang et al., 1994; Bellus et al., 1995; Tavormina et al., 1995). Mouse models of TD show cortical malformations, especially megalencephaly, however, without recapitulating the full pathophysiology (Lin et al., 2003; Inglis-Broadgate et al., 2005). *In utero* electroporation of fibroblast growth factor 8, a high-affinity ligand of fibroblast growth factor receptor 3, in the developing ferret neocortex successfully recapitulates the full cortical phenotype of TD, including polymicrogyria (Masuda et al., 2015). In this context, it is important to emphasize that such a folding defect, as a matter of principle, can only be recapitualted in a gyrencephalic, but not lissencephalic, animal model, which is the case for the ferret but not the mouse.

An important technical advance has been the creation of transgenic ferrets to model the cortical malformation due to mutation in *Aspm* (Johnson et al., 2018). First, whereas *Aspm* mutant mice exhibit only a mild microcephaly, *Aspm* knockout ferrets show severe microcephaly, with a 25–40% decrease in brain weight (Johnson et al., 2018). Second, whereas *Aspm* KO mice show a small reduction in cortical thickness, this parameter in *Aspm* KO ferrets, as in humans with mutations in *ASPM*, appears to be unaffected, with the major phenotype being a reduction in cortical surface area (Johnson et al., 2018). These phenotypes have been attributed to a large premature displacement of aRG to the oSVZ, implying a reduction in proliferative capacity of the displaced NPCs (Johnson et al., 2018).

These studies suggest that it would be interesting to use ferrets more extensively to study neocortical development, especially in the context of malformations, as they model human neocortical development more accurately than mouse. However, although ferrets are a superior model organism in this regard, they present several limitations, as follows. First, although the ferret brain is bigger than that of mouse and gyrified, it does not exhibit a prominent temporal lobe and lateral fissure (Hutchinson et al., 2017), two specific macroscopic features found in primate brains (Bryant and Preuss, 2018; Namba et al., 2019). Second, the available ferrets are outbred animals, and hence their genetic background is not homogeneous, in contrast to that of the inbred mouse lines. Third, the ferret genome annotation has not been completed yet, and therefore, for certain genes, gene manipulation is challenging. Fourth, the need of a special infrastructure for breeding ferrets, and the low number of pregnancies per year per animal, imply substantial financial investments when using ferrets.

### Non-human Primates

The closest animal models to human used in order to model neocortical malformations are non-human primates. Specifically, two non-human primates could be used to study neocortical development and to perform transgenesis, (i) the macaque, a gyrencephalic Old World monkey, and (ii) the marmoset, a near-lissencephalic New World monkey. A seminal study on neocortical development in fetal macaque (Betizeau et al., 2013) has provided insight into a number of key parameters, including bRG morphology. This has revealed that bRG morphology is more diverse (Betizeau et al., 2013) than originally assumed (Fietz et al., 2010; Hansen et al., 2010; Reillo et al., 2011). Very recently, transgenic macaques expressing the human *MCPH1* gene have been generated and reported to exhibit human-like neoteny of brain development (Shi et al., 2019).

The neocortex of the fetal common marmoset (*Callithrix jacchus*) exhibits an oSVZ with an abundance of bRG that is similar to that in the developing neocortex of gyrencephalic species (ferret, macaque, human). This observation underscores that bRG abundance alone is not sufficient to cause gyrencephaly (Garcia-Moreno et al., 2012; Kelava et al., 2012).

Marmosets were the first transgenic non-human primate to be established (Sasaki et al., 2009). As with transgenic macaques (Shi et al., 2019), transgenic marmosets could prove to be exceptionally useful animal models to provide insight into human neocortical malformations. However, generating transgenic non-human primates requires special animal facilities, is time-consuming and associated with very high costs. In the case of the marmoset, the gestation length of ≈150 days is a disadvantage when compared to the ≈40 days of ferret (Kawasaki, 2018). In addition, whereas the average litter size in ferret is 6–8 kits, it is only 1–2 in marmoset (Kawasaki, 2018). Importantly, the potential generation of transgenic non-human primates to model cortical malformations raises important ethical questions, especially if the experiments proceed beyond the gestational period.

### Cerebral Organoids

Recent advances in culture techniques using human induced pluripotent stem cells (iPSCs) and embryonic stem cells have allowed the establishment of brain organoids (Eiraku et al., 2008; Lancaster et al., 2013; Qian et al., 2016; Velasco et al., 2019). Such organoids are invaluable *in vitro* tools to study neocortex development and have provided insight into cortical malformations that involve alterations in progenitor cell behavior. Of note, cerebral organoids have been used to study the effects of Zika virus on the developing human brain and to screen for drugs that could potentially be used for treatment (Cugola et al., 2016; Dang et al., 2016; Nowakowski et al., 2016a; Qian et al., 2016; Wells et al., 2016; Xu et al., 2016; Watanabe et al., 2017; Zhou et al., 2017).

A key advantage of cerebral organoids is the option to use patient-derived iPSCs in order to model a given disorder. Thus, iPSCs derived from a patient suffering from severe microcephaly due to a *CDK5RAP2* mutation have been used to grow cerebral organoids. These organoids exhibited smaller neural tissue (Lancaster et al., 2013). Another study, using iPSCs from patients with mutations in the cadherin receptor ligand pair *DCHS1* and *FAT4*, has shown that cerebral organoids grown from these iPSCs recapitulate the cortical heterotopia observed in the patients, that is, a periventricular heterotopia and changes in progenitor cell morphology (Klaus et al., 2019). However, when using iPSCs from a patient, one should keep in mind that a somatic mutation that is responsible for a given cortical malformation may not be present in the blood cells or fibroblasts used to generate the iPSCs.

Another key advantage of cerebral organoids is that they provide a means of comparing neocortex development between primates (Mora-Bermudez et al., 2016; Otani et al., 2016; Pollen et al., 2019). Of note, cerebral organoids have been used to compare macaque cortical neurogenesis with chimpanzee and human cortical neurogenesis. Specifically, upper-layer neurons appeared earlier in macaque cerebral organoids than in chimpanzee and human cerebral organoids (Otani et al., 2016). Importantly, regarding the great apes, cerebral organoids are the only way to compare neocortex development between human and chimpanzee, gorilla, or orangutan. Indeed, while the cytoarchitecture of human and non-human primate cerebral organoids is very similar, comparison of cortical progenitor behavior in cerebral organoids derived from human and chimpanzee iPSCs has revealed a human-specific lengthening of metaphase during apical progenitor mitosis (Mora-Bermudez et al., 2016). With regard to human cortical malformations, it could be interesting to use cerebral organoids to compare a human microcephaly, caused by a gene mutation and resulting in the size of a chimpanzee brain, with a chimpanzee and to examine whether cortical progenitor behavior between the mutated human cells and the normal chimpanzee cells is similar or different.

Even if enormous progress has been made over last few years, it must be noted that human cerebral organoids are still far from being a perfect model for studying human brain development. Thus, the relative abundance of BPs, and in particular of bRG, is still low. Moreover, human cerebral organoids do not exhibit the cytoarchitecture observed in developing human neocortex, notably the iSVZ/oSVZ distinction. Also, the neuronal layering does not faithfully recapitulate the *in vivo* situation (Heide et al., 2018). Moreover, cerebral organoids display an upregulation of glycolysis (Pollen et al., 2019), which could be explained by the lack of oxygen supply to the center of the organoids. Cerebral organoids are not vascularized, and establishing a functional blood flow constitutes a major challenge. Folding is also a limitation of cerebral organoids, even if two recent studies have reported surface folding. One study reports folding in human 3D cerebral organoids upon *PTEN* deletion (Li et al., 2017); this folding likely reflects the activation of β-catenin, which can lead to folding of both apical and basal surfaces (Chenn and Walsh, 2002). The other study reports the appearance of wrinkles in human brain organoids using an on-chip approach (Karzbrun et al., 2018). Taken together, even if human cerebral organoids can recapitulate many hallmarks of human brain development, more research is needed in order to develop this exciting technology further.

## Conclusion

In order to study the pathophysiology of the various human cortical malformations, it is essential to use the appropriate experimental models. Mouse models have been most frequently used, and although they present numerous benefits, their limitations should be taken into consideration. Although most principles of the development of the neocortex are conserved across mammals, some important features are not present in mouse. Ferrets and non-human primates can be appropriate alternatives when a specific phenotype is not fully recapitulated in mouse. In the future, it will be essential to determine the role of bRG in the various cortical malformations, as mouse models do not allow studying these cortical progenitors. bRG are a hallmark of neocortex expansion in development and evolution, and a reduction in their abundance and proliferative capacity is likely to have drastic consequences on neocortex size. Importantly, further research on cerebral organoids appears to be crucial, as this technology holds many promises. Finally, while the research on the genetic causes of human neocortex malformations has so far been largely of academic and basic research interest, it is hoped that the knowledge gained will eventually open up new avenues, even at the fetal stage, to benefit the affected patients.

## Author Contributions

All authors wrote and edited the manuscript.

## Conflict of Interest Statement

The authors declare that the research was conducted in the absence of any commercial or financial relationships that could be construed as a potential conflict of interest.

## Abbreviations

aRG: apical radial glia
bIP: basal intermediate progenitor
BP: basal progenitor
bRG: basal radial glia
CP: cortical plate
GW: gestational week
INM: interkinetic nuclear migration
iPSC: induced pluripotent stem cell
iSVZ: inner subventricular zone
IZ: intermediate zone
MCPH: autosomal recessive primary microcephaly
NEC: neuroepithelial cell
NPC: neural stem/progenitor cell
oSVZ: outer subventricular zone
RG: radial glia
SVZ: subventricular zone
VZ: ventricular zone.
